# P-49. Clinical characteristics and treatment outcomes of Acinetobacter baumannii bloodstream infections in a setting with high carbapenem-susceptibility among isolates

**DOI:** 10.1093/ofid/ofaf695.278

**Published:** 2026-01-11

**Authors:** Jinghao Nicholas Ngiam, Matthew C Y Koh, Ka Lip Chew

**Affiliations:** National University Health System, Singapore, Singapore; National University Health System, Singapore, Singapore; National University Hospital, Singapore, Singapore, Not Applicable, Singapore

## Abstract

**Background:**

In most settings carbapenem-resistant *Acinetobacter baumannii* (CRAB) infections predominate over carbapenem-susceptible *Acinetobacter baumannii* (CSAB). Treatment guidelines focus on the management of CRAB and do not describe optimal antibiotic choice for CSAB. We describe clinical characteristics and outcomes in both CRAB and CSAB.

**Table 1:**
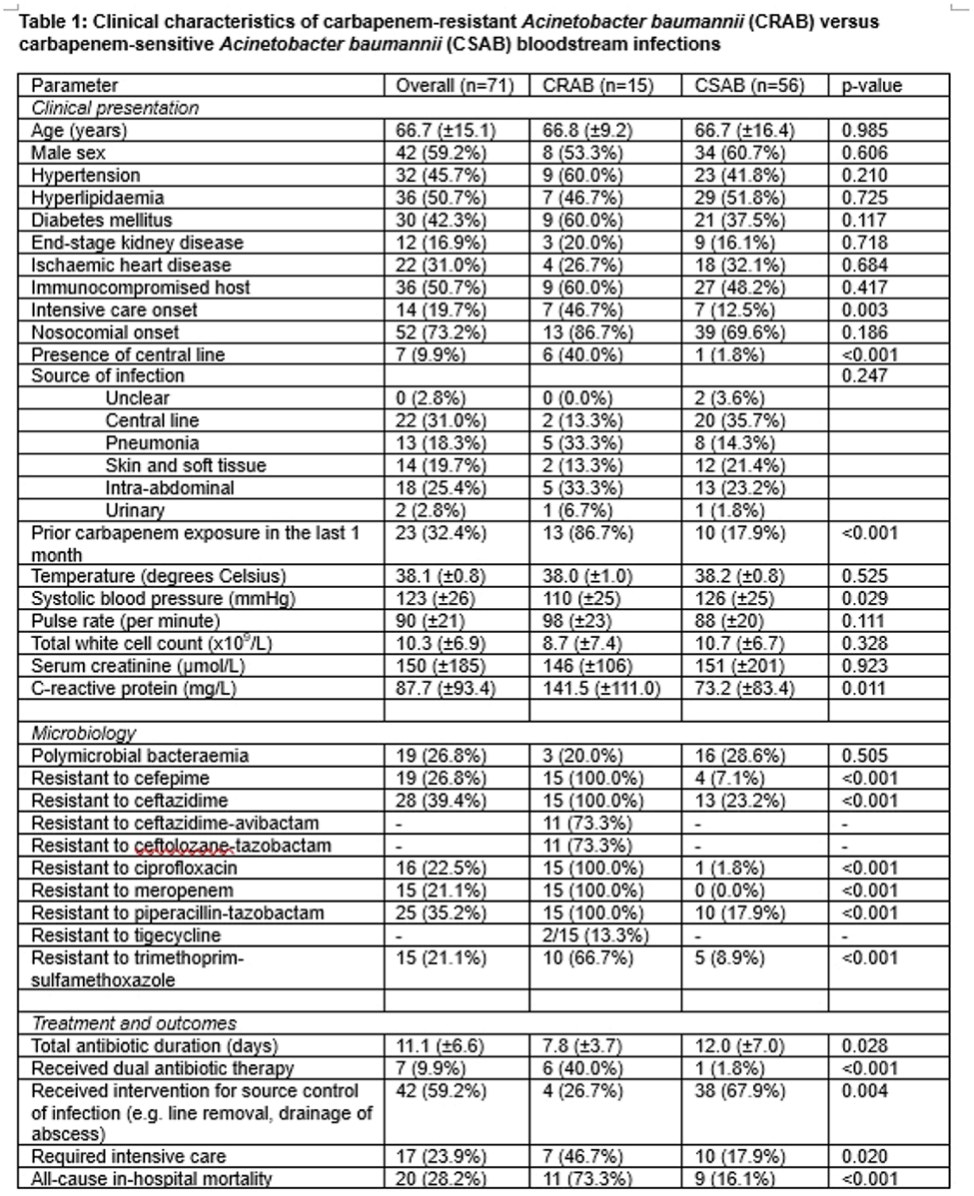
Clinical characteristics of carbapenem-resistant Acinetobacter baumannii (CRAB) versus carbapenem-sensitive Acinetobacter baumannii (CSAB) bloodstream infections

**Table 2:**
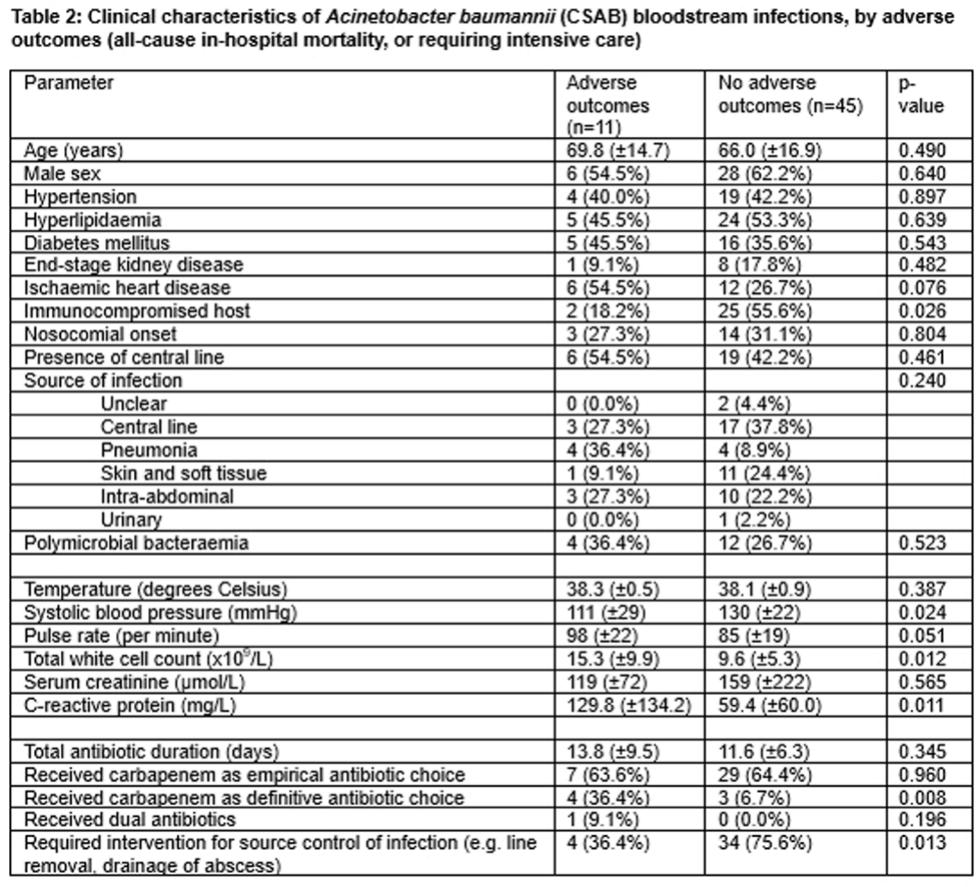
Clinical characteristics of Acinetobacter baumannii (CSAB) bloodstream infections, by adverse outcomes (all-cause in-hospital mortality, or requiring intensive care)

**Methods:**

We examined consecutive episodes of CRAB or CSAB bloodstream infections in our institution from February 2022 to July 2024. Clinical, laboratory and microbiological data were tabulated, and adverse outcomes were defined as the need for intensive care (ICU) or all-cause in-hospital mortality. We compared i) CRAB to CSAB, and amongst patients with CSAB, ii) examined the parameters associated with adverse outcomes by appropriate univariate and multivariable analyses.

**Results:**

We identified 71 unique episodes of bacteraemia, of which a majority 52/71 (73.2%) were nosocomial-onset. A minority of were CRAB (15/71, 21.1%). These patients often had ICU-onset, prior carbapenem exposure, and were more likely to experience mortality (73.3% vs 16.1%, p< 0.001). Only a minority of patients 7/71 (9.9%) received dual antibiotic therapy. Amongst the 56 patients with CSAB, only 11/56 (19.6%) experienced adverse outcomes. On multivariable analysis, after adjusting for elevated C-reactive protein, the use of carbapenems as the definitive antibiotic choice remained independently associated with adverse outcomes (adjusted odds ratio 7.34, 95%CI 1.25 – 43.01, p=0.027).

**Conclusion:**

CSAB was far more common than CRAB in our setting. Prior carbapenem exposure and ICU-onset were important risk factors for CRAB, which had higher mortality than CSAB. Carbapenem-sparing options may be considered as definitive antibiotic choice for CSAB.

**Disclosures:**

All Authors: No reported disclosures

